# Spatial and temporal disparities in general practitioner provision: a 21-year longitudinal analysis from Lower Saxony, Germany

**DOI:** 10.1186/s12942-025-00431-9

**Published:** 2025-12-04

**Authors:** Jonas Schoo, Frank Schüssler

**Affiliations:** Department of Civil Engineering Geoinformation Health Technology, Jade University of Applied Sciences, Oldenburg, Germany

**Keywords:** General practitioner care, Spatial inequality, Rural health, Longitudinal trend analysis, Lower Saxony, Germany, Healthcare planning, Urban–rural divide

## Abstract

**Background:**

Equitable access to general practitioner services remains a persistent challenge for health systems and is critical for reducing health inequalities, particularly between urban and rural regions. Understanding the spatial and temporal dynamics of primary care provision is vital for informed healthcare planning and policy.

**Methods:**

Spatial and temporal disparities in the supply of general practitioners across Lower Saxony, Germany, were assessed over a 21-year period (2000–2021). Data from the Association of Statutory Health Insurance Physicians and municipal population statistics were used to develop the General Practitioner Regional Index - a composite measure incorporating both the availability and accessibility of general practitioners. Non-parametric statistical tests were applied to identify significant trends at the municipal level.

**Results:**

Pronounced geographic inequities in general practitioner supply were identified. More than 40% of the population resides in areas with declining supply, while only 5% experience improvements. Urban centres and their peripheries consistently exhibited higher and mostly stable levels of general practitioner supply. In contrast, rural small towns and villages demonstrated both lower baseline accessibility and more frequent negative trends. The primary driver of supply losses in affected areas was physician retirement, while relocation played a secondary role and typically occurred within similar regional types, thereby limiting redistribution effects.

**Conclusions:**

The results underscore the persistence of urban–rural disparities in general practitioner availability and highlight physician retirements as the principal factor behind declining supply, with limited offsetting effects from physician migration. The findings indicate a need for spatially sensitive, succession-focused workforce strategies and innovative primary care models to mitigate rural undersupply and promote health equity.

**Trial registration:**

Not applicable. This study does not report the results of a health care intervention on human participants.

## Introduction

Ensuring equitable access to general practitioner (GP) services remains a critical challenge for health systems worldwide [1, 2]. GPs play a key role in prevention, care coordination, and chronic disease management [3–6]. Unequal access intensifies health disparities, particularly between urban and rural areas [7, 8]. In Germany, rural or structurally weak regions consistently face shortages of GPs, negatively affecting healthcare accessibility and outcomes [9–14]. Spatial and socioeconomic factors - such as settlement patterns and area deprivation - strongly influence healthcare utilisation. Residents in rural and disadvantaged areas need longer travel distances to GPs and lower service use, highlighting spatial inequities that demand targeted policy responses [3, 9, 11, 15]. Germany expects a shortage in GP workforce: in 2019 approximately one-third of GPs were 60 years or older, and projections estimate a deficit of around 20,000 GPs by 2025 due to retirements exceeding the number of new GPs entering practice [16]. In the federal state of Lower Saxony, data from 2024 show that 37,1% of GPs being over 60 years of age [17]. According to used data, the average retirement age has increased from just over 53 in 2000 to more than 62 in 2021 [18]. As a consequence, the growing number of retirements has increasingly led to permanent closures of particularly rural practices and mounting workload stress for ageing physicians who continue practicing [4]. In Lower Saxony, about one in six GP positions could be unfilled by 2035, particularly in rural and northern as well as western regions, highlighting the significant challenges in maintaining adequate healthcare provision in the coming years [17].

In response to these challenges, a range of structural reforms have been suggested, such as binding service definitions (i.e., legally required minimum portfolios of GP services that must be offered by all practices), expanding multiprofessional care centres, broader use of non-physician health professionals, and regulated specialist training quotas [16]. Germany has a centrally defined needs-based planning framework for ambulatory physician care, but physicians retain a certain degree of freedom in choosing both their specialty and their preferred practice location inside planning areas. As a result, the combination of national planning guidelines together with individual choice, can make it difficult to implement uniform workforce policies and fully correct regional imbalances in GP supply [16]. To address these persistent challenges at the regional level, both the Lower Saxony Ministry for Social Affairs, Employment, Health and Equal Opportunities and the Association of Statutory Health Insurance Physicians of Lower Saxony have jointly developed a comprehensive 10-point action plan to strengthen general practitioner care in the state [19]. This action plan builds on the national planning framework but introduces additional region-specific initiatives targeting medical education, training, and practice conditions. Central components include the expansion of medical study places, support for rural placements, facilitation of lateral entry into general practice, reduction of administrative burdens, and the promotion of telemedicine and multiprofessional primary care centres. By bundling these measures with regular progress reviews, the plan aims to address both short- and long-term shortages in GP supply and improve healthcare accessibility across urban and rural regions of Lower Saxony [19].

A key prerequisite for effective policy is the accurate assessment of GP supply, not only in terms of availability but also spatial accessibility [20, 21]. While planning frameworks regulate supply at aggregated supra-local levels, recent research has identified substantial gaps between planning targets and actual [22], fine-scale access, particularly in rural and some urban regions [15, 23]. These studies employ a cross-sectional approach and primarily focus on analysing the current distribution of general practitioners in Germany while investigation on temporal trends is rarely found with the exception of developments in China between 2010 and 2019 [24] and in Germany from 2015 to 2019 [3]. Thus, research on small-area temporal trends in GP accessibility in Germany and other contexts remains limited.

To address this gap, this study suggests a new spatially sensitive measure - the General Practitioner Regional Index (GPRI).

To provide an initial assessment of disparities in GP provision, the Gini coefficient [25–29] was calculated for the GPRI values from 2000 to 2021 which has been applied in international health research [30–32]. The Gini summarizes overall inequality across municipalities, offering a first indication of the sources of inequality. Finally, robust longitudinal trend analyses over a 21-year period at the municipal level in Lower Saxony were conducted. Furthermore, to examine whether regional disparities in GP accessibility exhibit spatial clustering, the study also applies measures of spatial autocorrelation using global and local Moran’s I. This allows for the identification of spatially coherent patterns of under- or oversupply that extend beyond simple inequality measures. This approach yields systematic data that can inform needs-oriented and regionally balanced primary care provision.

By combining the GPRI with these inequality metrics, longitudinal trend analyses, and spatial autocorrelation measures, this study captures both the level, spatial structure, and temporal dynamics of regional disparities in primary care supply.

This study therefore addresses the following research questions:

- How has GP supply changed across Lower Saxony from 2000 to 2021?
- How do these trends vary among different spatial typologies, from urban to rural areas?
- Which workforce-related factors, such as retirement or relocation, contribute to observed changes?

The analysis focuses on Lower Saxony, a federal state in north-western Germany, covering approximately 47,700 square kilometres and home to around 8.1 million inhabitants [33, 34]. The state exhibits pronounced spatial heterogeneity, with around 58% of the population living in urban areas and 42% in rural areas [35]. This diversity in settlement structure, combined with substantial differences in regional healthcare provision, makes Lower Saxony particularly suitable for analysing spatial and temporal disparities in general practitioner availability.

In 2023, the needs-based planning classification identified 1.45% of the population as living in undersupplied areas, 40.44% in imminent undersupply, 41.04% in regular supply, and 17.07% in oversupplied areas. An alternative analysis using the 2SFCA method at street-segment level yielded 25.26% in undersupplied areas, 24.82% in imminent undersupply, 10.04% in regular supply, and 39.88% in oversupply. These figures demonstrate that general practitioner provision in Lower Saxony is highly heterogeneous across the population [22]. Furthermore, Lower Saxony was selected due to the availability of high-quality, longitudinal municipal-level data spanning 2000 to 2021, including information on general practitioner provision, population structure, and settlement characteristics. Such detailed, consistent data are not uniformly accessible across all German federal states. Although comparable information might be available from other Associations of Statutory Health Insurance Physicians, obtaining and harmonising data from all regions would be highly demanding. Limiting the analysis to one federal state is therefore a pragmatic and methodologically sound approach. While the analysis focuses on Lower Saxony as a case study, the findings are of broader relevance: the patterns of spatial heterogeneity observed reflect challenges in balancing healthcare supply across urban and rural areas, which are common in many countries [36–41]. In this sense, Lower Saxony serves as an example for understanding regional disparities in primary care provision in other national or international contexts.

## Background

The needs-related planning guideline (Bedarfsplanungsrichtlinie) in Germany, set by the Federal Joint Committee (Gemeinsamer Bundesausschuss, G-BA), regulates the number and spatial distribution of statutory health insurance (SHI) accredited general practitioners [42, 43]. GP planning areas, which can encompass one or more municipalities, are used to calculate target GP numbers based on the local population and a morbidity adjustment [43, 44]. Actual GP numbers are compared with these targets to classify areas as undersupplied, imminently undersupplied, regularly supplied, or oversupplied, determining eligibility for new practice licenses. Physicians must obtain a SHI practice position (Kassensitz) from the Association of Statutory Health Insurance Physicians (KV) to treat and bill for SHI patients. While the guideline ensures nationwide outpatient care, physicians’ freedom to choose a practice location within a planning area can create local disparities, potentially resulting in oversupply or undersupply on a small scale within specific municipalities [42].

Until the amendment of the guideline in 2012, ten different population-to-physician ratios applied across Germany, which were linked to ten types of planning areas as defined by the Federal Office for Building and Regional Planning. This meant that regional differences in GP supply were, to some extent, predetermined and cannot be interpreted as indicators of poor provision. With the reform of 2012, planning areas for GP care were restructured, shifting from districts and independent cities to so-called medium-sized areas (Mittelbereiche), of which 883 exist nationwide. These areas represent the functional catchment areas of at least lowest order central places, thereby aiming to achieve a more balanced distribution of GPs. Since 2012, state-level planning authorities have also been able to deviate from the nationally given area types and ratios, for example by merging small Mittelbereiche or subdividing large cities into districts [45].

At the same time, analyses at the municipal level remain valuable, as they capture fine-grained spatial differences that may not be visible at higher levels of aggregation. In the present study, this issue is addressed by using the General Practitioner Regional Index, which explicitly incorporates cross-boundary supply effects.

The GPRI is a descriptive, accessibility‑oriented measure intended to capture the availability and spatial reachability of general practice over time. It is methodologically independent of the German needs‑based planning framework and neither evaluates nor replicates its criteria or determinations. The index is used to analyse the historical development and small‑area patterns of GP provision, irrespective of planning areas or thresholds. Accordingly, GPRI values are not directly comparable with planning grades or designations of under‑ or oversupply and should not be interpreted as such; rather, they provide complementary empirical evidence to inform, but not assess, formal planning.

## Data

Multiple data sources were used to analyse the distribution and trends of GP supply in Lower Saxony from 2000 to 2021. This timeframe ensures consistent availability of demographic, geographic, and healthcare data.

GP location data were obtained from the *Association of Statutory Health Insurance Physicians Lower Saxony*, covering the years 2000 through 2021. Access was authorized by the *Ministry of Social Affairs*,* Employment*,* Health*,* and Equal Opportunities of Lower Saxony*, complying fully with data protection regulations [18].

Population statistics at the municipal level were sourced from the INKAR database, published by the *Federal Institute for Research on Building*,* Urban Affairs and Spatial Development* for the same period, complemented by municipal boundary data from the Federal Agency for Cartography and Geodesy [46–48]. To approximate residents’ spatial distribution within municipalities, historical ATKIS (*Authoritative Topographic-Cartographic Information System*) land use data were employed, extracting residential building areas and mixed-use areas for each year. The ATKIS is Germany’s official topographic geospatial database, maintained by the *Federal and State Cartographic Agencies*, providing highly detailed and standardised information on land use and settlement structures nationwide. Centroids of these areas served as spatial proxies for residential locations [46].

To differentiate settlement structures relevant for healthcare supply analysis, municipalities were classified according to the RegioStaR 5 spatial typology developed by the *Federal Ministry for Digital and Transport*. This typology distinguishes five spatial categories: metropolis, urban region, urban periphery, rural urban areas (larger suburban or small-town communities), and rural small towns and villages (more peripheral and sparsely populated areas). Incorporating these typologies enables a nuanced stratified analysis of GP accessibility across diverse spatial contexts [35].

Overall, data quality was consistently high with no significant gaps or missing values, supporting robust temporal and spatial data analyses.

## Methods

To analyse temporal trends in GP supply, the study developed the GPRI. It is based on the Health Resources Density Index (HRDI) which is designed to incorporate both demographic factors and geographic dimensions in the assessment of healthcare resource distribution [24, 49]. While the HRDI effectively integrates area and population, it does not fully capture two critical aspects of healthcare accessibility: (1) the reachability of healthcare providers within a municipality and across municipal borders, and (2) the actual distance residents must travel to see a GP. Functional reachability considers not only the presence of providers but also their availability to the local population, consisting of geographic barriers and in population distribution within the area. Actual travel distance accounts for the spatial separation between residents and providers, which directly influences timely access to GPs. Together, these dimensions affect healthcare utilization and patient outcomes beyond what simple provider-to-population ratios can indicate [50].

It should be noted that while the Two-Step Floating Catchment Area (2SFCA) method and related gravity-based approaches are widely regarded as best practice for accessibility analyses in health geography, their application was not feasible in the present study [22, 51–53]. The 2SFCA requires detailed historical road network data and small-area population distributions to accurately model travel times and provider-to-population ratios within catchment areas. Such data were not consistently available for the study period, precluding the use of these methods. Consequently, the GPRI was developed as a pragmatic alternative, combining population-to-GP ratios and straight-line travel distances to approximate spatial accessibility. Although the GPRI does not fully capture the interaction effects modelled in 2SFCA, it provides a robust, longitudinally consistent measure of GP availability and reachability across municipalities given the limitations of historical data.

Accessibility in this study is operationalised as the reachability of general practitioners, measured by (a) the average distance from residential points to the nearest GP and (b) the ratio of inhabitants in a municipality to the number of GPs in a municipality as well as the average number of GPs within a 9.17 km radius of each residential point. This measure goes beyond simple distance to capture the potential availability of multiple GPs within a reasonable travel range. It should be noted, however, that other dimensions of accessibility, such as contract coverage with statutory health insurance, opening hours, or service type (private/public), are not considered in this analysis due to lack of consistent data. Consequently, the GPRI reflects geographic accessibility rather than actual utilisation or service access.

The GPRI extends the HRDI by integrating two key components reflecting GP accessibility and availability at the municipal level:

- Availability Component (Ratio of population to GP numbers, both, directly within the municipality and as reachable from residential locations within it): For each year and municipality, the average number of reachable GPs per residential and mixed-used location (derived from ATKIS data) was calculated by counting GPs within a 9.17 km radius of straight-line distance - corresponding to a 10-minute driving time per car at 55 km/h. The 9.17 km threshold follows common practice in healthcare accessibility studies, which define adequate access as a 10–20 min car travel time to a GP [54]. National data confirm generally short travel times: according to the *Infrastrukturatlas Deutschland*, 94.7% of residents reach a medical practice within 5 min [55], and the *Deutschlandatlas* reports median travel times of 4 min and 89% of the population within 5 min [56]. The assumed average speed is based on a closest-facility analysis (street section points to GP locations) conducted in 2023 for Lower Saxony, using detailed road network data and the maximum legally permitted speed limits. This analysis yielded a mean effective driving speed of 50.3 km/h, with 55 km/h adopted in the model to represent ideal, congestion-free travel conditions. To assess the robustness of this assumption, sensitivity analyses were conducted using alternative travel scenarios. A 20-minute threshold at an average speed of 41.6 km/h resulted in a correlation of *r* = 0.998 with the baseline scenario, while a 10-minute threshold at 35 km/h (corresponding to a radius of 11.67 km) produced an almost identical correlation of *r* = 0.99996. In addition, a scenario based on the empirically observed average effective driving speed of 50.3 km/h and a 15-minute travel time (≈ 12.58 km radius) yielded a correlation of *r* = 0.99994. These findings confirm that the normalization procedure within the GPRI formulation effectively captures relative spatial disparities while minimizing the influence of the absolute reachability threshold, ensuring the robustness of the index across plausible parameter variations. This approach captures cross-municipal reachability, i.e., the potential for residents to access GPs in neighbouring municipalities.
- Accessibility Component (Average Euclidian distance from residential locations to the nearest GP): The average distance from each residential centroid to the nearest GP was computed, reflecting real-world travel considerations. Due to the lack of historical road network data, straight-line distances were used as validated proxies, showing a strong positive correlation (*r* = 0.8) with actual driving times in 2023. For each municipality and year, the arithmetic mean of all residential centroid-to-nearest GP distances was calculated to obtain a single representative accessibility measure.

The formula for the GPRI is defined as follows:

$$\begin{aligned}\:\text{GPR}{\text{I}}_{\text{iy}} & =\left[1-\text{Nor}{\text{m}}_{\text{min-max}}\left(\sqrt{\frac{{P}_{iy}}{{G}_{iy}}+\frac{{P}_{iy}}{{R}_{iy}}}\right)\right]\\ & \quad \cdot\:\left[1-\text{Nor}{\text{m}}_{\text{log}\text{-min-max}}\left({D}_{iy}\right)\right] \end{aligned}$$

where:

$GPRI_{iy}:$ *the value of the GPRI in the ith municipality in year y,*

$\:{P}_{iy}:\:$ *population of the ith municipaliy in year y,*

$\:{G}_{iy}:\:$ *number of GPs in the ith municipality in year y,*

${R}_{iy} :\:$ *average number of reachable GPs per residential point in the ith municipality in year y,*

${D}_{iy} :\:$ *average distance to the nearest GP per residential point in the ith municipality in year y,*

$\text{Nor}{\text{m}}_{\text{min-max}} :\:$ *Min-max normalization over all municipalities and years,*

$\text{Nor}{\text{m}}_{\text{log}\text{-min-max}}:$ *Log-transformed min-max normalization over all municipalities and years.*

The GPRI ranges from 0 (extreme undersupply with high population load per GP, low reachability, and large distances) to 1 (optimal availability and accessibility with low population load, many reachable GPs, and short distances).

The GPRI is calculated based on population size, GP numbers, average distance to the nearest GP, and the average number of reachable GPs from each residential point. It should be noted that the index does not adjust for population morbidity or socio-economic characteristics. These factors were not included in the needs-based planning guideline prior to 2019, and their exclusion in the present analysis reflects the historical planning framework.

Distributional equality was quantified using the Gini coefficient (formular see appendix), a widely applied measure derived from the Lorenz curve. The coefficient denotes the proportion of the area between the Lorenz curve and the line of complete equality relative to the total area beneath that line, and takes values from 0 (perfect equality) to 1 (complete inequality) [57, 58]. Both, unweighted and population weighted Gini coefficients were calculated. To evaluate temporal trends in the GPRI at the municipality level, several non-parametric statistical methods were employed. Formulas can be found in the appendix.

- The Mann-Kendall test is a statistical method used to detect monotonic trends in a time series without requiring the data to conform to any specific distribution [59, 60]. It was computed using annual GPRI values from 2000 to 2021 to identify positive, negative, or no trends in GPRI. The analysis outputs included the z-value, indicating trend direction and strength, and the p-value, assessing statistical significance (*p* < 0.05 denotes a significant trend) [61]. The Mann-Kendall test is suitable for this analysis for several reasons. First, it is robust to non-normal data and outliers, making it appropriate for GPRI values that range between 0 and 1 and may contain abrupt local changes [62]. Second, it remains valid even in the presence of autocorrelation or scaling effects in the data, which can otherwise increase Type-I error rates (false detection of trends) [63].
- To complement the Mann-Kendall Index, the Theil-Sen Estimator was calculated for each municipality for GPRI values. This estimator determines the slope of a trend in a time series and is particularly resistant to the influence of outliers [64–66]. The resulting Theil-Sen-Slope represents the median annual change in GPRI values. Positive slope values indicate an increase in healthcare resource density, while negative values suggest a decline. The Theil–Sen estimator is well-suited for analysing GPRI trends because it is a non-parametric method that calculates the median of all pairwise slopes, providing robust trend estimates [67, 68]. It is resistant to outliers and does not assume a specific data distribution [68], making it appropriate for GPRI values between 0 and 1 and heterogeneous municipal-level data.
- Kendall’s Tau, a rank correlation coefficient, was computed to quantify the strength and direction of the association between GPRI values and time. It is closely tied to the Mann-Kendall test and provides additional insights [65, 69]: Tau coefficient measures correlation on a scale from − 1 to 1, where − 1 indicates a strong negative correlation (GPRI decreases over time), 1 indicates a strong positive correlation (GPRI increases over time), and 0 signifies no correlation [70]. Kendall’s Tau is well-suited for analysing GPRI trends because it is a non-parametric rank correlation coefficient that measures the strength and direction of monotonic trends without assuming a specific data distribution. This makes it robust to outliers and appropriate for GPRI values ranging between 0 and 1. By standardizing trends between − 1 and 1, Kendall’s Tau enables consistent comparison of trends across municipalities [71, 72].

Since the statistical methods applied are designed to detect monotonic trends in the data series, abrupt changes within a single year were sometimes not identified as statistically significant. Therefore, an additional change point detection was conducted, which allowed for the recognition of structural breaks and significant shifts that may otherwise have been missed. For each municipality, two trend analyses were conducted:

1. An overall trend over the entire 2000–2021 period;
2. A series of rolling trends (six-year window).

Trend classification combined four statistical indicators - p-values, z-values, Kendall’s Tau, and Theil-Sen slope according to the following criteria as shown in Table 1.

**Table 1:** Classification categories for trend analysis

Trend	*p*- | z- values	Kendall’s Tau	Theil-Sen slope
**Strong negative trend**	*p* < 0.05, |z| >1.96	Tau < 0	Theil-Sen < −0.015
**Moderate negative trend**	*p* < 0.05, |z| >1.96	Tau < 0	Theil-Sen < −0.005
**Weak negative trend**	*p* < 0.05, |z| >1.96	Tau < 0	Theil-Sen < 0
**No statistical trend**	*p* ≥ 0.05	--	--
**Weak positive trend**	*p* < 0.05, |z| >1.96	Tau > 0	Theil-Sen > 0
**Moderate positive trend**	*p* < 0.05, |z| >1.96	Tau > 0	Theil-Sen > 0.005
**Strong positive trend**	*p* < 0.05, |z| >1.96	Tau > 0	Theil-Sen > 0.015

The thresholds for the Theil–Sen slope were determined based on the observed distribution of slope values across municipalities, and practical significance. Slopes below 0.005 are considered weak, representing minor changes over the 21-year period. Moderate slopes (≥ 0.005) correspond to a cumulative change of approximately 10.5% (0.005 × 21 ≈ 0.105 on a 0–1 scale), reflecting noticeable changes in GP availability. Strong slopes (≥ 0.015) represent substantial changes, amounting to roughly 31.5% over the study period (0.015 × 21 ≈ 0.315). This classification allows trends to be interpreted as both statistically robust and substantively meaningful, in line with applications of the Theil–Sen estimator in environmental and human geography.

This classification ensures that identified trends are both statistically significant and substantively meaningful in magnitude, facilitating robust identification of municipalities with notable increases or decreases in GP supply over time.

To assess the presence of spatial clustering in municipal GPRI trends, both Global Moran’s I and Local Moran’s I were computed. Global Moran’s I provides a single summary measure of overall spatial autocorrelation across the study area, whereas Local Moran’s I decomposes this relationship to identify individual municipalities that contribute significantly to spatial clusters of high or low values. A positive Moran’s I indicates spatial clustering of similar values, a negative Moran’s I suggests spatial dispersion, and a value near zero implies a random spatial distribution [73, 74].

Local Moran’s I provides a measure of the degree to which a municipality’s value is similar to that of its neighbours. The statistical significance of is assessed using permutation tests, allowing identification of high–high, low–low, high–low, or low–high spatial associations [73, 75]. A non-significant Global Moran’s I indicates that GPRI trends are not spatially structured at the overall level, although Local Moran’s I can still reveal spatially coherent subregions or outliers.

All statistical analyses and data management were performed using RStudio (Version 2024.04.2) using the packages *dplyr*, *ggplot2*, *Kendall*, and *trend*. Geoprocessing was conducted with ArcGIS Pro (ESRI) (Version 3.5.0).

## Results

This section examines the state of GP supply at the municipal level in 2021 and its inequality, long-term trends across Lower Saxony. The relationship between current GP supply and these temporal trends was assessed, including stratification by regional typology (RegioStaR). Finally, for municipalities experiencing negative trends, the underlying causes are explored by distinguishing between GP retirements and relocations, and by identifying the destination of migrating physicians.

### Spatial patterns of GP supply in 2021

**Fig. 1:**
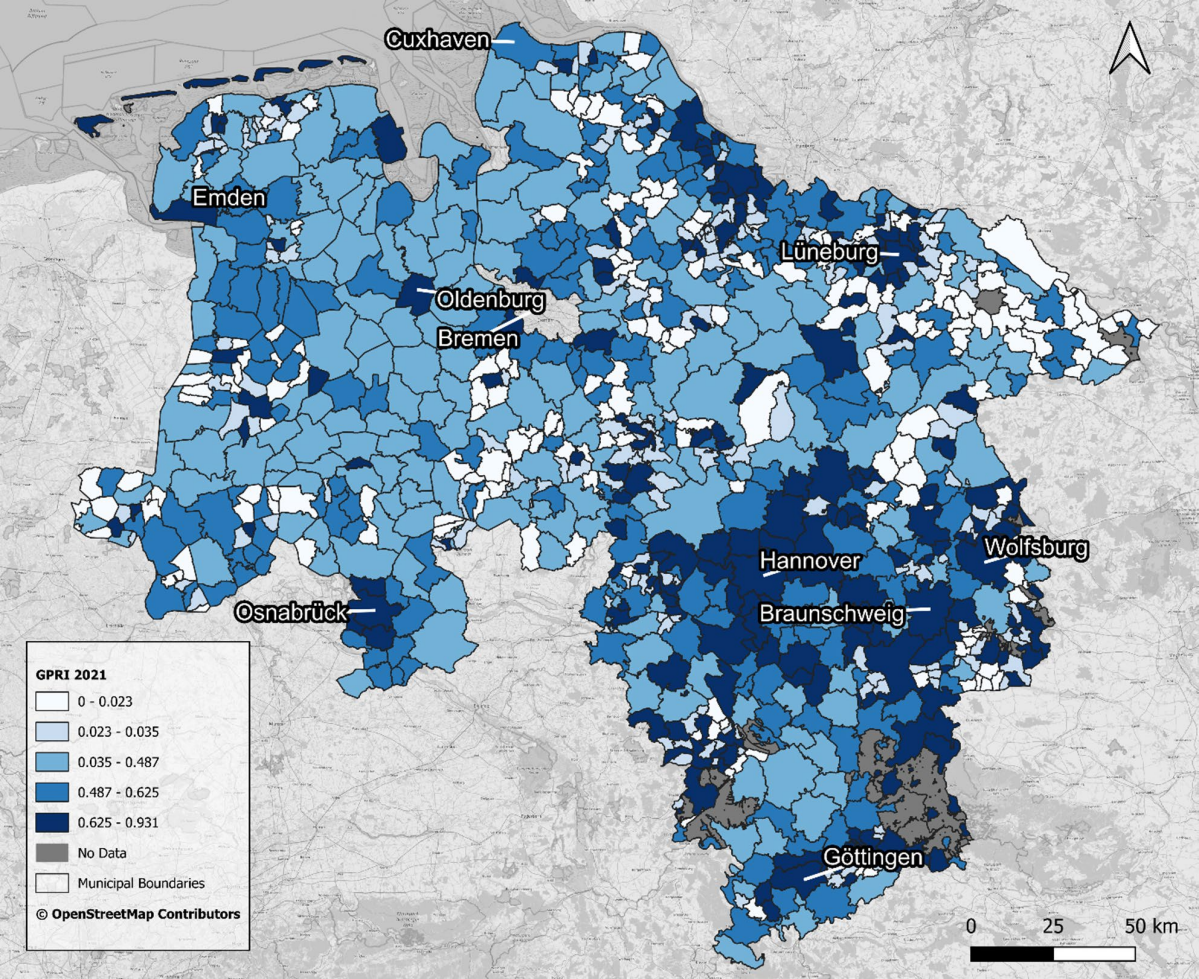
GPRI 2021 in Lower Saxony at municipality level. Data: [18, 47, 48]

Figure 1 shows the GPRI values at the municipality level for 2021. The median GPRI is 0.43, with a mean of 0.35 (SD = 0.29), ranging from 0 to 0.93. This distribution highlights considerable variability in GP provision across municipalities, with some areas clearly underserved. The class intervals are based on quantiles to ensure an even distribution of municipalities across classes and to facilitate comparability of relative differences.

Municipalities in the southern, south-eastern, and eastern regions - particularly in the Göttingen area, the Hannover region, and the Braunschweig and Wolfsburg regions - exhibit higher GPRI values, indicating better overall GP supply. Additionally, all major cities including Osnabrück, Oldenburg, Emden, Delmenhorst, Göttingen, Braunschweig and Hanover as well as the Lower Saxony islands, show similarly strong GPRI values, reflecting robust GP supply. Conversely, municipalities in the northwest, such as those in Wesermarsch, Cloppenburg, and parts of the Cuxhaven area, demonstrate lower to medium GPRI values. Notably, in the northeast near the Lüneburg area, GP supply appears to be comparatively poor. The central part of Lower Saxony exhibits considerable variability, with neighbouring municipalities showing markedly different levels of GP supply.

### Inequality of GPRI (2000–2021)

**Fig. 2:**
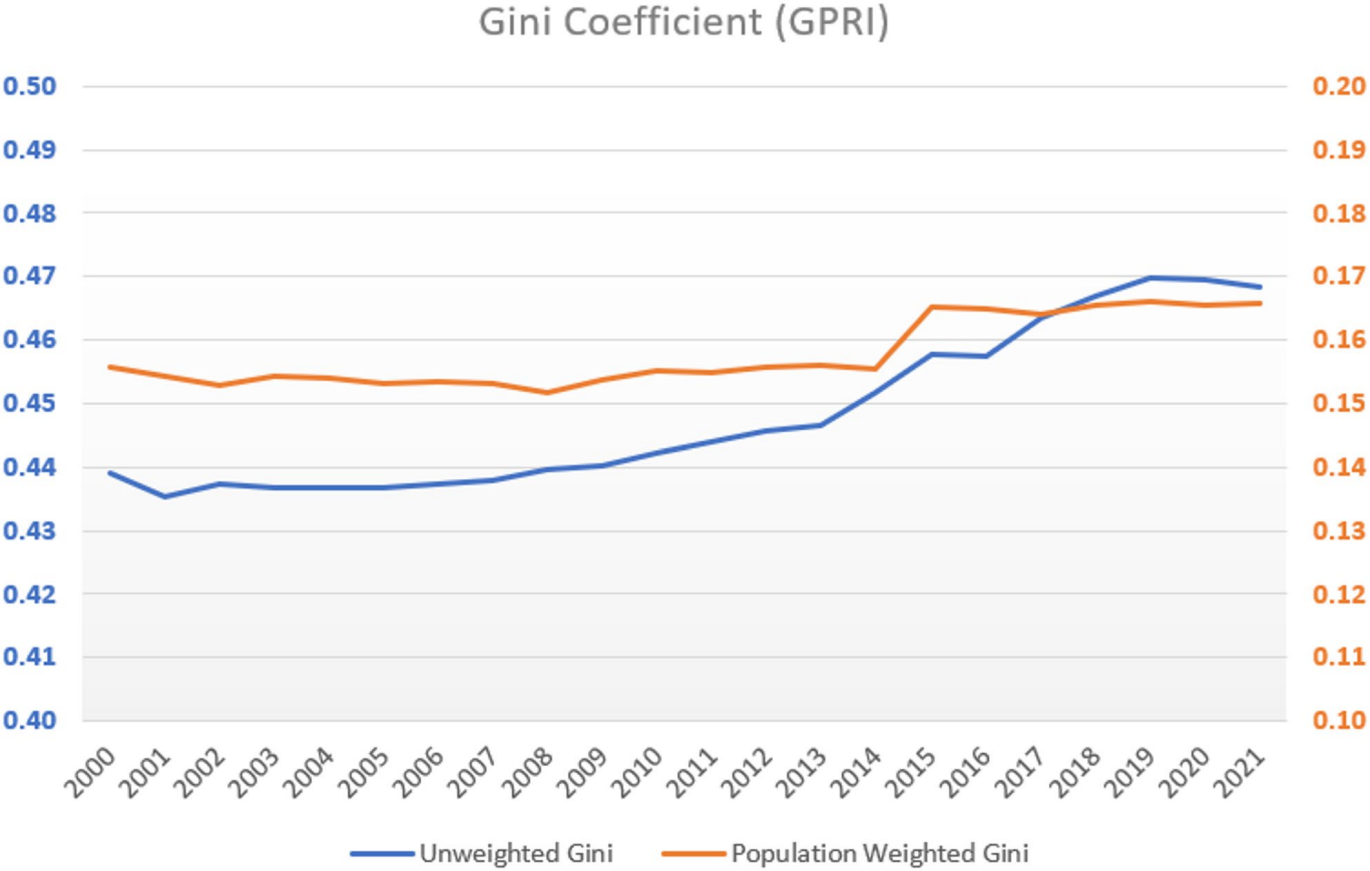
Gini Coefficients of GPRI values from 2000–2021

To assess disparities in GP accessibility across municipalities in Lower Saxony, both population-weighted and unweighted Gini coefficients were calculated for the period 2000–2021. The population-weighted Gini remained consistently low, ranging from 0.1529 in 2002 to 0.1661 in 2019 (Fig. 2). The slight upward trend indicates that disparities in GP accessibility increased only minimally over time, with most of the population continuing to live in municipalities with relatively good GP coverage.

The unweighted Gini, which treats each municipality equally regardless of population size, ranged from 0.435 to 0.470, also showing only a very small increase over the study period. Overall, the comparison between weighted and unweighted Gini coefficients demonstrates that while minor variations exist, the magnitude of inequalities across municipalities remained largely stable. These findings provide a rationale for the subsequent longitudinal analyses using the GPRI, which allow the temporal and spatial dynamics of GP accessibility to be examined in detail.

### Long-Term trends in GP supply (2000–2021)

**Fig. 3:**
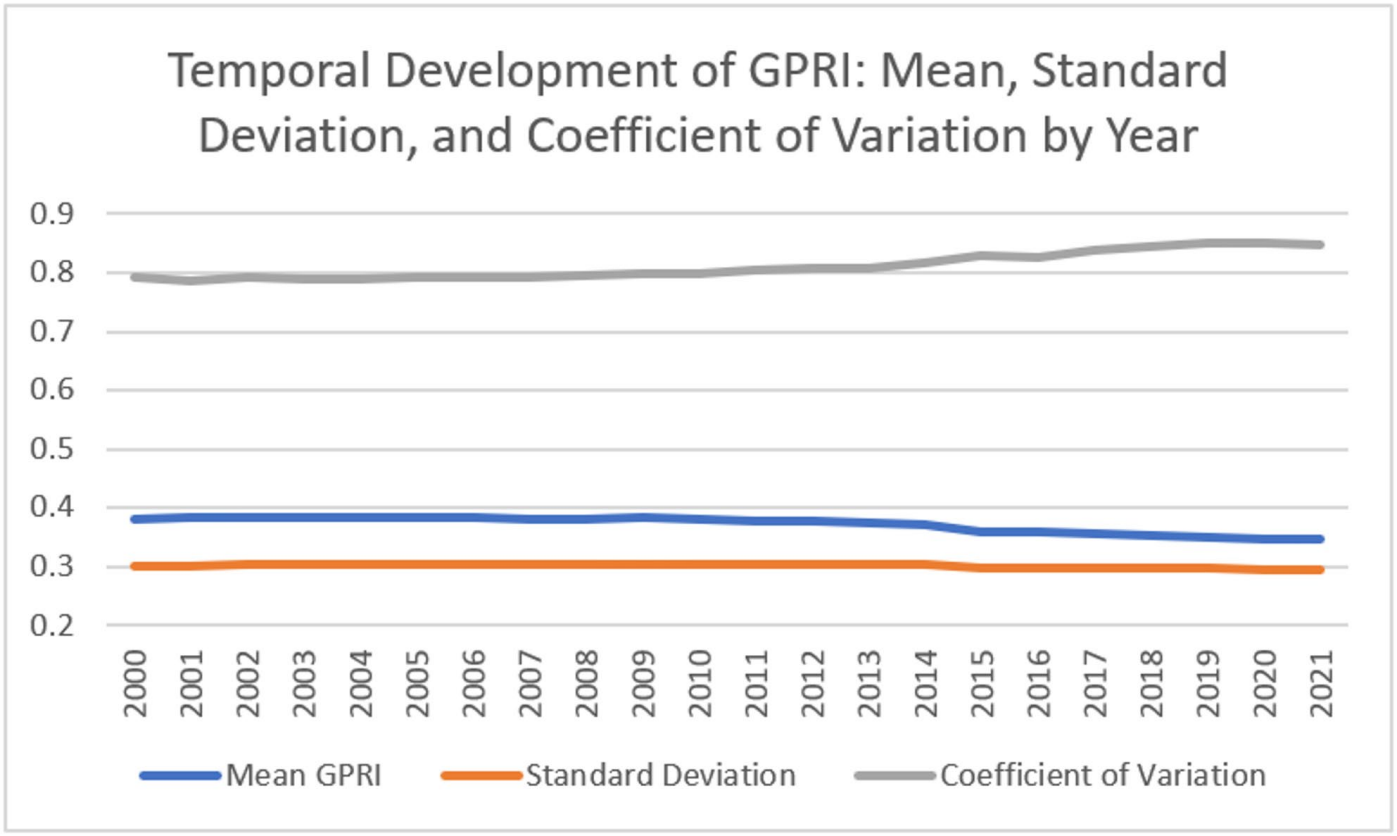
Mean GPRI, standard deviation, and coefficient of variation of the GPRI across municipalities in Lower Saxony from 2000 to 2021

Figure 3 illustrates long-term trends in GP supply across Lower Saxony from 2000 to 2021, showing the mean GPRI, its standard deviation, and the coefficient of variation, which together highlight both the overall accessibility and the extent of variability between municipalities over time.

Over the period 2000–2021, the mean GPRI across municipalities in Lower Saxony declined slightly from 0.380 to 0.384 in the early 2000 s to 0.347–0.346 by 2021, indicating a modest decrease in average GP accessibility. The standard deviation remained relatively stable around 0.29–0.30, suggesting that the absolute variation in GPRI between municipalities did not change substantially. However, the coefficient of variation increased from 0.79 to 0.85, signalling that relative inequalities in GP accessibility between municipalities grew slightly over time, even as overall accessibility remained fairly consistent.

**Fig. 4:**
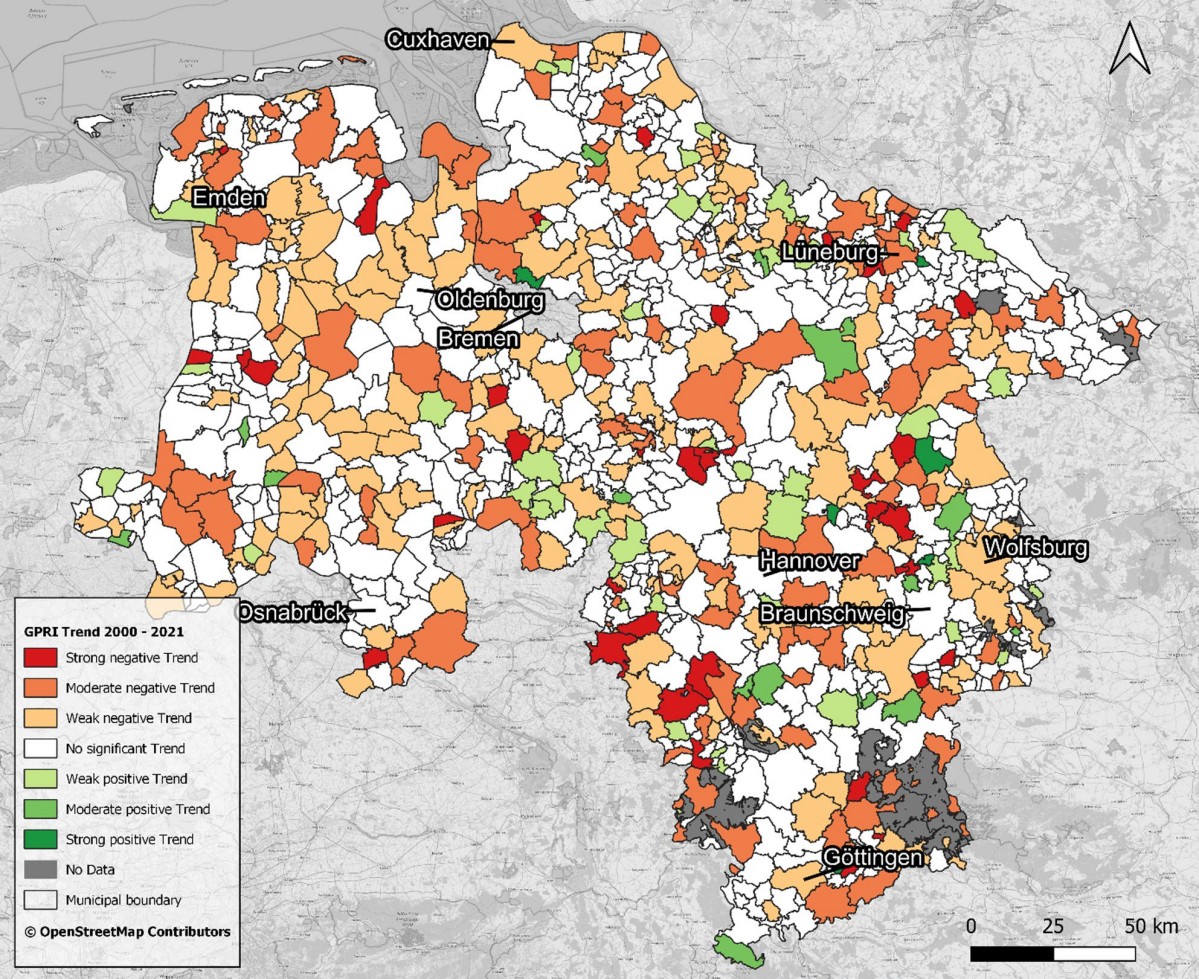
Statistical Trend of the GPRI in Lower Saxony from 2000 to 2021 on the municipality level. Data: [18, 47, 48]

Figure 1 reveals distinct clusters of municipalities with similar levels of current GP supply across Lower Saxony; however, Fig. 4 - which illustrates statistical trends in GPRI from 2000 to 2021 at the municipal level, categorised by trend direction and magnitude - reveals a much more heterogeneous spatial pattern, reflecting diverse dynamics of change across the region. Out of 941 municipalities, the majority (57.6%) exhibited no significant trend. Negative trends were more frequent than positive ones: 18.7% showed weak negative, 12.1% medium negative, and 4.0% strong negative trends. Positive trends were less common, with 5.3% weak positive, 1.6% medium positive, and 0.6% strong positive. This distribution highlights that while many municipalities maintained relatively stable GP accessibility, a substantial minority experienced notable increases or declines over the study period.

Municipalities with increasing, decreasing, and non-significant GPRI trends are often found adjacent to each other, resulting in a highly heterogeneous and fragmented pattern. This coexistence of different trend classes is especially evident in central Lower Saxony, where clear, continuous areas of similar trends are largely absent. A comparison across the RegioStaR 5 [35] spatial typology highlights urban–rural disparities: over 70% of residents in metropolitan and urban areas experienced stable or mildly negative GPRI trends, whereas 15% of residents in rural small towns and villages experienced moderate or strong negative trends, with an additional 14% experiencing weak negative trends. Positive developments were rare (< 2%) in these rural areas. Overall, more than 40% of the population resides in municipalities with worsening GP supply, while only 5% reside in areas with improving trends.

Global spatial autocorrelation of municipal GPRI trends, measured by Moran’s I (I = 0.026, z = 1.54, *p* = 0.12), was not statistically significant, indicating that increases or decreases in GPRI were spatially unstructured. In other words, municipalities with similar temporal trends in GPRI did not form distinct regional clusters. Consistent with the non-significant global Moran’s I, local Moran’s I did not reveal strong or spatially coherent clusters of municipalities with similar GPRI trends.

### Combined analysis: current supply and trends

**Fig. 5:**
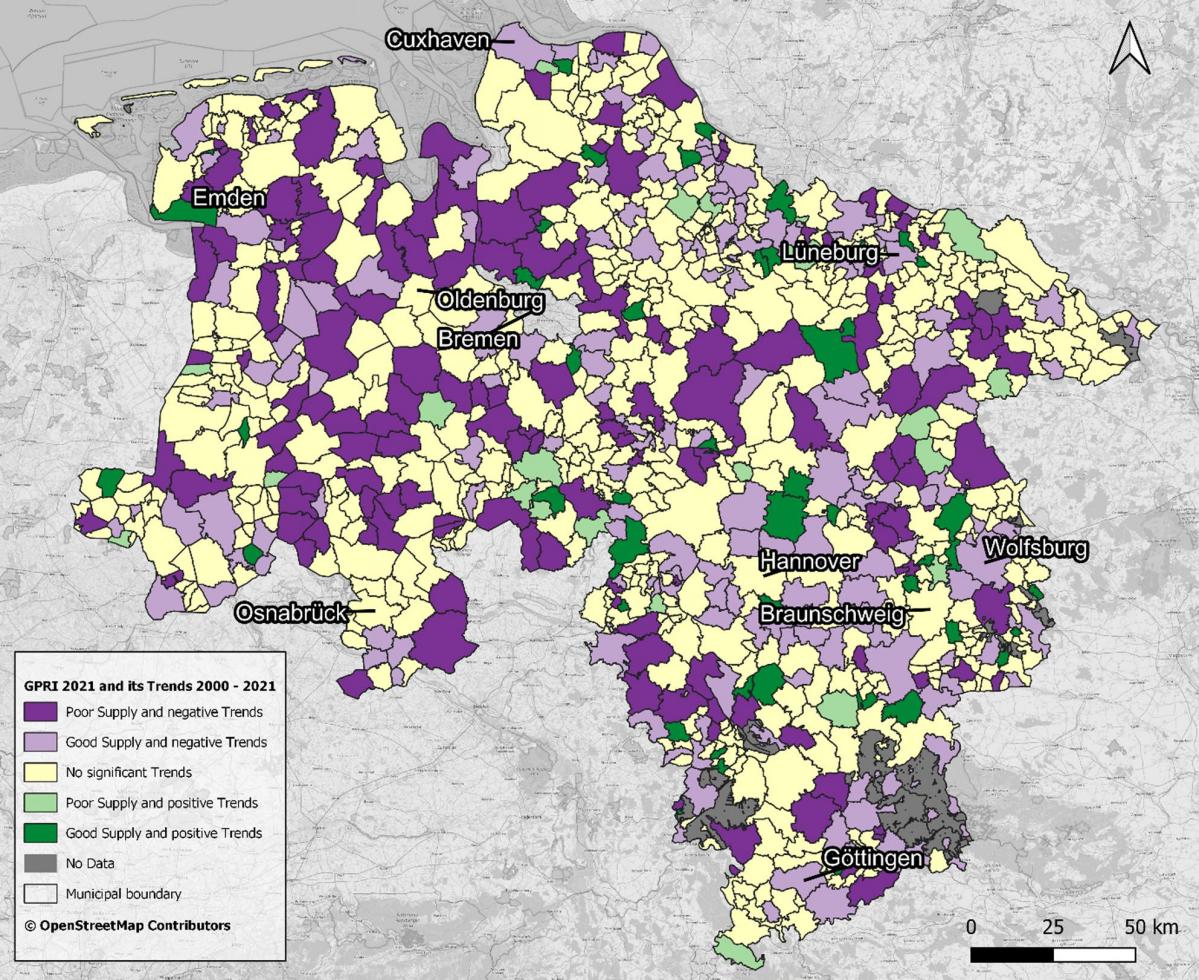
GP Supply in 2021 and Long-Term Trends (2000–2021) on municipality level. Data: [18, 47, 48]

In 2021 concentrated urban centres and surrounding suburbs like Hanover, Braunschweig, Osnabrück, and Göttingen showed high GP supply, whereas peripheral rural areas - especially in the northwest and parts of the northeast and south - exhibited lower GP supply. Long-term trends from 2000 to 2021 did not reveal consistent spatial patterns, showing a heterogeneous and fragmented distribution of changes in GP supply across municipalities. This lack of clear geographical clustering contrasts with the spatial disparities observed in the GP supply (2021), the trends do not align neatly with the existing spatial distribution of GP supply. Central urban areas generally showed stable GP supply, but the overall pattern indicates a complex and non-uniform evolution of GP supply. Correspondingly, Fig. 5 combines GP supply in 2021 and trend directions, highlighting large areas in western and central Lower Saxony with both below average supply and negative, worsening trends, contrasted by few clusters of well-supplied municipalities with positive developments in the east. Areas with stable supply but no clear trend, especially in the north-east, show that developments have been mixed. Here too, the proximity of different classes highlights the overall heterogeneity of the spatial pattern.

Quantitative analysis of GPRI trends, combined with the RegioStaR settlement typology, reveals distinct differences in both baseline GP supply and trend across Lower Saxony as shown in Table 2.

**Table 2:** Distribution of population by GPRI trend and GPRI 2021 across regiostar Spatial typologies

GPRI and Trends	Poor supply and negative trends	Good supply and negative trends	No significant trend	Poor supply and positive trends	Good supply and positive trends
Metropolis	0%	0%	100%	0%	0%
Urban region	0%	34%	66%	0%	0%
Urban periphery	9%	37%	50%	0%	4%
Rural urban area	12%	35%	45%	2%	7%
Rural small town/villages	26%	19%	47%	3%	5%

Stable or improving GP supply is largely limited to metropolitan and urban regions, where most residents experience no significant trend or stable supply. In contrast, negative developments and persistent undersupply dominate in rural and peripheral areas. Particularly in rural small towns and villages, stability is much less common, and a substantial share of the population is exposed to both poor supply and further declines. These findings highlight clear regional differences, with rural populations disproportionately affected by worsening GP supply. Population based mean values reveal that 14% of Lower Saxony’s inhabitants face poor supply and negative Trends, 28% good supply and negative Trends, positive Trends are rare with a total of 5%. The majority experiences no significant trend with a percentage of 54%.

### Relationship between GP supply and trends

To complement these findings on regional differences in GP supply, the relationship between supply levels in 2021 and long-term trends were evaluated. Correlation analysis indicates no relationship (*r* = 0.054), suggesting current GP supply levels do not predict historical increases or decreases. Municipalities with high or low GPRI in 2021 do not have consistently followed matching trends.

**Fig. 6:**
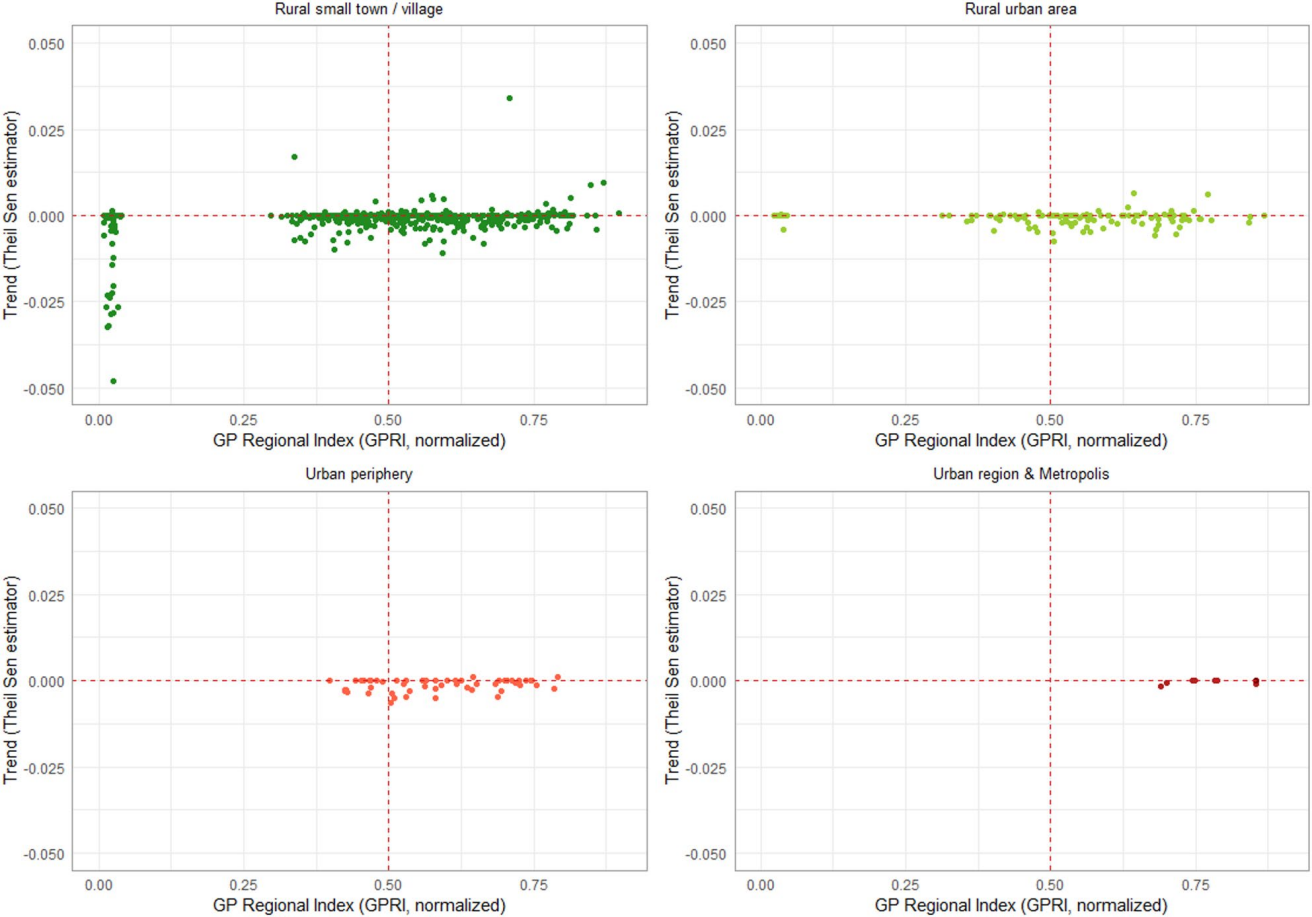
Scatterplot of GP Supply in 2021 and Long-Term Trends (2000–2021) by Regional Typology 5 on municipality level

To further examine the relationship between GPRI in 2021 and its trends, scatterplots were generated comparing the GPRI values to the trends using the Theil-Sen estimator stratified by RegioStaR 5 typology. To focus on statistically significant trends, only municipalities with significant Theil-Sen estimator (α = 0.05) were included in Fig. 6.

The upper left quadrants show municipalities with below-average GP supply but improving trends, indicating potential recovery or recent improvements in supply. The upper right quadrants represent municipalities with above-average GP supply and a positive trend, suggesting areas with both strong supply and positive trends. In contrast, the lower left quadrants include municipalities with both low GP supply and negative trends, reflecting the most vulnerable areas in terms of primary healthcare supply. The lower right quadrants comprise municipalities with relatively good GP supply but declining trends.

**Rural small towns and villages:**

- Most pronounced disparities and compounded disadvantages in GP supply.
- Significant share of municipalities have both low current GP supply and declining trends.
- 64 outlier municipalities with extremely low supply and strong negative trends; none of these had a practising GP in 2021, no spatial pattern recognisable.
- These outliers are scattered across Lower Saxony and all share very small population sizes (~ 400–4,000, average ~ 1,500).
- To explore socio-demographic characteristics of the 64 municipalities identified as GPRI outliers, a comparison with all other municipalities using data from 2001/2013 to 2021, as well as absolute changes over time was computed. Variables included total population, unemployment, mean age, net migration, household income, purchasing power [47], and the German Index of Socioeconomic Deprivation (GISD) [76]. Given that most variables were not normally distributed (Shapiro–Wilk test, *p* < 0.05 for several variables), group comparisons were performed using Mann–Whitney U tests. Mean and median values were calculated for each variable and group.
- Key patterns emerged: Outlier municipalities were consistently smaller than non-outliers (*p* < 0.001). Although population changes over time were somewhat lower in outliers, these differences were not statistically significant. Regarding unemployment, outlier municipalities historically exhibited lower unemployment rates (*p* < 0.001); however, the increase over time was significantly higher (*p* = 0.0004), indicating a relative worsening of the employment situation. The mean age of the population was initially slightly lower or comparable in outliers, but by 2021 these municipalities had an older population, and the increase in mean age over time was significantly greater (*p* = 0.00066). No significant group differences were observed for net migration. Similarly, purchasing power and the proportion of low-income households differed only slightly and mostly non-significantly between groups, although a minor increase over time was evident in outliers. No significant differences were found in the GISD, suggesting that socio-economic deprivation did not consistently distinguish outlier municipalities. Overall, GPRI outliers tended to be smaller, older, and experienced stronger increases in unemployment and population aging, while other socio-economic indicators showed no clear or significant patterns.

**Rural urban areas:**

- Display a more concentrated pattern, with most municipalities clustered around the median for GP supply and near-zero trends.
- Negative developments are present but less severe and more scattered than in smaller rural settlements.

**Urban peripheries:**

- Generally, exhibit favourable conditions for GP supply.
- Most municipalities are located to the right of the median line, indicating good supply and trends that are mostly stable or slightly negative.

**Urban/metropolitan regions**:

- Show the most advantageous healthcare supply situation.
- Data points are densely clustered, reflecting both high and stable levels of GP supply.
- Trends in these regions are predominantly stable or slightly positive.

Although the overarching trends largely mirror the urban-rural gradient - where urban areas tend to have more stable or favourable developments and rural areas face greater challenges - a small number of municipalities deviate from these patterns. These limited exceptions - such as rural areas with improving trends or urban areas with slight declines - demonstrate that categorising municipalities as ‘urban’ or ‘rural’ does not fully determine the direction of GP supply and its trends. Instead, these cases highlight the significance of local factors and targeted interventions, which may lead to developments that differ from the broader urban–rural pattern.

Hence, Fig. 6 reinforces the earlier finding of a spatially polarized healthcare landscape. Rural regions are not only more likely to face lower levels of GP coverage but also disproportionately affected by declining trends. In contrast, urban areas show higher resilience and more favourable development trajectories.

### Determinants of negative trends: retirement and relocation

To better understand the drivers behind the decrease of GP supply in municipalities exhibiting strong or moderate negative GPRI trends, the proportional contributions of physician retirement and physician relocation were analysed by RegioStaR category.

**Table 3:** Share of retirement and relocation in GP supply decline by regiostar typology

RegioStaR 5	Retirement	Relocation
Urban periphery	65%	35%
Rural urban area	63%	37%
Rural small town/villages	71%	29%

As seen in Table 3, retirement is consistently the main driver negative trends in GP supply across the regional types, particularly in smaller and rural municipalities. Relocation plays a comparatively smaller role, especially in rural small towns and villages. When aggregating across all categories, 69% of supply reductions were linked to retirement. Overall, the data highlight that workforce aging is the dominant driver of supply decline, while migration of GPs is more relevant in urban or peri-urban contexts.

To further address the destination of physicians leaving their municipalities, an origin-destination matrix was analysed to track migration flows between the RegioStaR spatial categories.

**Table 4:** Migration flow of gps in municipalities with moderate or strong negative trends by regiostar category

	GPs moving from…		
GPs moving to…	From Rural small town/village	From Rural urban areas	From Urban periphery
To Rural small town/village	54%	12%	12%
To Rural urban areas	14%	50%	7%
To Urban periphery	20%	31%	72%
To Urban region	11%	2%	7%
To Metropolis	0%	5%	2%

Table 4 reveals that most GP relocations occur within the same regional typology, indicating that GPs tend to move locally rather than between fundamentally different spatial typologies. However, there is a slight tendency for movement from less urbanised areas - particularly rural small towns and villages and rural urban areas - towards more urbanised locations, especially the urban periphery, with approximately 20% of GPs from rural small towns relocating there. The visible migration of GPs from rural to central urban areas emphasises the importance of retaining doctors in vulnerable rural communities. Ongoing retirements pose the greatest threat to sustaining adequate healthcare coverage.

### Overall results

In conclusion, following results can be summarised:

Research Question 1: How has GP supply evolved across Lower Saxony from 2000 to 2021?

The GP supply in Lower Saxony over the 21-year period has been heterogeneous. Inequality analyses using Gini coefficients indicate that disparities increased slightly over time. Statistical trend analysis shows that while urban and metropolitan areas maintained relatively stable or even slightly improving supply, rural and peripheral areas exhibited more frequent and pronounced declines. The observed trends are characterized by multiple waves of deterioration, especially notable in rural municipalities during the periods 2013–2018 and 2014–2019. Overall, the average GP density showed a slight decline, and these patterns reveal complex temporal and spatial variability in GP supply.

Research Question 2: How do these trends vary among different spatial typologies?

Global spatial autocorrelation of GPRI trends, measured by Moran’s I, was not statistically significant, and local Moran’s I did not reveal coherent local clusters, suggesting that temporal trends are largely spatially unstructured, however, differences become apparent when considering spatial typologies. Rural areas - particularly small towns and villages - are disproportionately stronger affected by declines in GP supply. In municipalities with negative trends, approximately 14% of the population lives in areas with low GP supply and worsening conditions. This contrasts with urban and metropolitan areas, where supply tends to be higher and more stable, with limited occurrence of negative trends. Trends in peri-urban and rural urban municipalities are somewhat intermediate, with moderate levels of decline and stability. The highly fragmented spatial distribution of trends indicates that disparities persist and that rural areas bear the brunt of deteriorating GP availability.

Research Question 3: Which workforce-related factors, such as retirement or relocation, contribute to observed changes?

Workforce dynamics analysis revealed that the majority of reductions in GP supply, about 69%, can be attributed to retirements, while 31% result from relocations. Notably, most GP relocations occur within the same regional typology, with a slight tendency for movement from rural small towns and rural urban areas towards urban peripheries. Migration from rural to central urban areas remains limited. This emphasises that retirements are the principal driver of supply reductions, particularly in vulnerable rural and peripheral regions.

## Discussion

This study provides comprehensive insights into the spatial and temporal dynamics of GP supply in Lower Saxony over a 21-year period. The findings reveal disparities between urban and rural areas, with urban centres and their peripheries demonstrating relatively stable or improving GP coverage, while rural and peripheral regions face significant challenges marked by both low baseline supply and ongoing declines. These observations are consistent with previous research highlighting persistent rural healthcare supply [9, 16, 22] and its trends [3].

Recent evidence from several high-income countries highlights persistent disparities in the distribution of primary care staff. In England, Nussbaum et al. (2021) observed that over time, GPs, paramedics, and other direct patient care staff have become increasingly underrepresented in socioeconomically deprived areas, whereas physician associates and pharmacists are comparatively more prevalent. These trends suggest that workforce inequalities are not static but have widened in some cases, reinforcing the “Inverse Care Law,” whereby healthcare provision is lowest where health needs are greatest [39]. Similarly, US data from 2009 to 2017 show that although the overall density of primary care physicians, nurse practitioners, and physician assistants increased in rural counties, growth in urban counties was faster, thereby exacerbating rural–urban gaps [77]. In France, GP density decreased between 2007 and 2017, while territorial disparities increased. GPs are more likely to locate in cantons with existing healthcare infrastructure and higher quality-of-life indicators, whereas cantons with fewer services or lower integration into metropolitan regions experience slower growth or stagnation in GP numbers. The establishment of multiprofessional health centres has partially offset these shortages in underserved areas, particularly in peri-urban and rural fringes, by attracting young practitioners and allowing shorter working hours [78].

The predominance of physicians’ retirements as the main driver of declining GP supply aligns with demographic trends of an ageing medical workforce [3, 4, 16]. The fact that only a minority of supply reductions is explained by physician relocation raises questions about the limited effectiveness of natural workforce redistribution in addressing regional shortages. Furthermore, the tendency for GPs to move within similar regional typologies, with relatively little migration from rural to urban cores, suggests that current mobility patterns largely maintain existing regional disparities, as they do not entail significant movement from underserved to better supplied regions.

The critical challenge identified by van den Bussche (2019) - namely, a projected shortage of up to 20,000 GPs in Germany by 2025 due to retirements outpacing new entrants - is strongly corroborated by current data and analysis. The KVN forecasts that the number of general practitioners in Lower Saxony will decrease from around 5,200 today to approximately 3,750 by 2035, leaving about one in six GP positions unfilled. This underprovision is expected to be particularly severe in rural regions, where extended travel times to practices and more frequent closures due to a lack of successors are already observed. As van den Bussche argues, this trend cannot be explained by the number of medical graduates alone but is also linked to a growing preference for specialty training and changes in work patterns, such as increased part-time employment and salaried contracts - particularly among younger and female doctors. The KVN also points out that, due to these developments, 1.6 new GPs are now required to replace every retiring doctor in terms of working hours. The high average age of the current medical workforce further exacerbates the situation [17].

Both van den Bussche and stakeholders stress the urgency of comprehensive reforms that go beyond merely increasing the number of study places. Necessary approaches include modernizing and making general practice training more attractive and flexible, implementing effective management tools such as the rural GP quota, providing financial and organizational support for setting up practices, and intensifying the delegation of non-physician tasks. The KVN has launched a range of support mechanisms in line with these recommendations, including financial incentives for practice foundations, career change incentives, scholarships for students, income guarantees for new practices, and targeted support for training physicians and rural internships [19, 79].

No single intervention suffices for recruitment and retention in underserved regions; rather, a coordinated set of strategies is necessary. Given the overwhelming role of physician retirements, short-term policies such as financial and regulatory incentives (targeting group practice, rural training programs, or location-based bonuses) may be paired with long-term investments in the medical workforce. This includes integrating rural rotations into curricula, encouraging applicants with rural backgrounds, and improving working conditions through task delegation and more flexible work arrangements [80].

Recent German survey data indicate that biographical and family-related factors play a major role in GPs’ practice location choices. In addition, psychosocial attitudes - such as a strong focus on patient-centred care - are particularly characteristic of those who choose to work in primary care, distinguishing them from physicians who pursue other specialties [81]. This is also reflected in recent large-scale surveys among German GPs, which emphasize concerns about working conditions, professional identity, and the perceived effectiveness of current policy measures [6]. However, such criteria have recently gained more attention in German policy discussions. For instance, the introduction of the rural doctor quota, expanded medical school places in Lower Saxony, and initiatives to strengthen general practice in medical curricula represent important steps toward more targeted recruitment for underserved regions [16, 82]. While structured rural training programs and targeted selection are already part of ongoing reforms in several federal states, they are not yet broadly implemented nationwide as in some other countries [16].

Beyond workforce planning, innovative care models such as multiprofessional primary care centres, the HÄPPI model [83], and the introduction of community-based health kiosks may mitigate shortages by redistributing tasks and expanding service reach. These approaches are supported by a growing integration of non-physician health professionals and increased use of digital health and telemedicine, offering greater flexibility and accessibility, particularly for rural areas [4, 9].

To effectively address these challenges, health workforce planning may integrate granular socioeconomic data and adapt dynamically to temporal changes. Spatial and longitudinal modelling approaches, such as those utilised in this study, could provide promising tools for such adaptive planning [3, 84]. Importantly, recent evidence underlines the need to incorporate GP perspectives more systematically in the design and implementation of workforce and primary care reforms [6].

Strengths of this study include the use of high-quality data and detailed geospatial information, enabling robust spatial and temporal analyses. However, limitations must be acknowledged: the assumption of a 9.17 km radius for accessibility approximates travel, but may not reflect individual travel routes or perceptions; straight-line distances may introduce imprecision; travel chains and personal accessibility perceptions are not captured; cross-border interactions are largely ignored; and accessibility in municipalities without any GPs may exaggerate perceived undersupply. Additionally, unmeasured factors such as patient and physician preferences could not be considered due to data constraints. Finally, another limitation of this study relates to changes in the institutional framework of needs-related planning. Until the amendment of the Bedarfsplanungsrichtlinie in late 2012, ten different population-to-physician ratios and planning area types were in effect. As a result, some observed regional disparities in GP provision may reflect regulatory differences rather than solely variations in actual care provision. From 2013 onwards, the shift to medium-sized areas (Mittelbereiche) and the introduction of a single ratio across Germany fundamentally altered the planning logic. For this reason, trends observed before and after 2013 are not fully comparable, and the interpretation of pre-2013 disparities should be made with caution. Moreover, municipalities located at the borders of Lower Saxony may have access to GPs located in neighbouring federal states such as Bremen or Hamburg. Due to data constraints, these cross-state co-supply effects could not be fully captured in the GPRI calculation. In addition, the allocation of statutory physician seats (Kassensitze) is carried out by the respective regional Associations of Statutory Health Insurance Physicians (Landes-KVen), which may also influence local GP availability. As a result, accessibility in border municipalities may be underestimated or overestimated, and findings for these areas should therefore be interpreted with caution.

Furthermore, the analysis does not incorporate population morbidity or socio-economic characteristics. Nevertheless, future studies could integrate morbidity and socio-economic factors to provide a more refined assessment of local healthcare demand.

The GPRI offers several advantages over traditional measures of GP availability, including the HRDI and the widely used 2SFCA method. While the HRDI considers provider-to-population ratios, it does not fully capture spatial distribution, and the 2SFCA requires detailed road networks, historical traffic, and small-area population data, which are often unavailable for long-term studies.

In contrast, the GPRI integrates availability and accessibility by combining population-to-GP ratios with spatial measures, including the average distance to the nearest GP and the number of GPs reachable within a defined radius. It captures cross-municipal reach, is robust to outliers, and can be calculated consistently over multiple years, enabling reliable longitudinal analyses of trends and inequalities in GP provision. Its pragmatic data requirements - municipal population, GP locations, and residential points - make it feasible even without detailed historical infrastructure data. By explicitly incorporating accessibility and cross-boundary supply effects, the GPRI allows a consistent comparison across municipalities over time, providing fine-grained spatial differentiation that highlights disparities not visible with conventional density-based measures.

Overall, the GPRI represents a practical, robust, and spatially sensitive tool for monitoring GP accessibility and trends, complementing existing indices while overcoming their respective limitations in data availability and spatial resolution.

## Conclusion and outlook

This study highlights significant spatial and temporal disparities in general practitioner supply across Lower Saxony, particularly between urban and rural areas. While differences observed before 2013 were partly influenced by the needs-based planning guideline, these patterns also reflect real variations in accessibility and availability, which persisted independently of regulatory frameworks. From 2013 onwards, standardised planning allows for a more consistent assessment of genuine disparities in provision. The findings emphasise that physician retirements are the primary driver of declining GP availability, with limited compensatory effects from migration. Addressing these challenges requires a multifaceted approach, including reforms in medical education, targeted recruitment incentives, and the expansion of innovative care models such as multiprofessional centres and telemedicine. Importantly, future analyses should integrate measures of service need - including population age structure, morbidity, and socioeconomic vulnerability - as these factors critically shape both the demand for and the accessibility of primary care. Accounting for service need would allow a more nuanced assessment of spatial disparities and better align planning frameworks with actual healthcare requirements. Future workforce planning should also integrate detailed socioeconomic data and dynamic spatial analyses to effectively guide policies aimed at ensuring equitable and sustainable primary care provision. Further research could usefully explore the perspectives and proposed solutions of key stakeholders - including policymakers, needs-based planning authorities, patient representatives, and medical associations - to identify actionable strategies and foster broad consensus for addressing regional disparities in GP supply.

## Appendix

### Appendix I – Gini coefficient

The Gini coefficient is defined as follows [57, 58]:

$$\:Gy=\frac{2{\sum\:}_{i=1}^{{n}_{y}}i\hspace{0.17em}GPR{I}_{i,y}}{{n}_{y}{\sum\:}_{i=1}^{{n}_{y}}GPR{I}_{i,y}}-\frac{{n}_{y}+1}{{n}_{y}}$$

$$\begin{aligned}\:{G}_{y}^{\left(w\right)} &=\frac{2\sum\:\text{i}={1}^{{\text{n}}_{\text{y}}}\text{i},{\text{P}}_{\text{i},\text{y}},\text{G}\text{P}\text{R}{\text{I}}_{\text{i},\text{y}}}{{\sum\:}_{\text{i}=1}^{{\text{n}}_{\text{y}}}{\text{P}}_{\text{i},\text{y}}{\sum\:}_{\text{i}=1}^{{\text{n}}_{\text{y}}}\text{G}\text{P}\text{R}{\text{I}}_{\text{i},\text{y}}} \\ & \quad -\frac{{\sum\:}_{\text{i}=1}^{{\text{n}}_{\text{y}}}{\text{P}}_{\text{i},\text{y}}+1}{{\sum\:}_{\text{i}=1}^{{\text{n}}_{\text{y}}}{\text{P}}_{\text{i},\text{y}}} \end{aligned}$$

where:

${G}_{y}:\:$unweighted Gini Coefficient in year y,

${G}_{y}^{\left(w\right)} :\:$population weighted Gini Coefficient in year y,

${GPRI}_{i,y} :\:$the value of the GPRI in the ith municipality in year y,

${P}_{i,y}:\:$population of the ith municipaliy in year y.

### Appendix II – Mann-Kendall test

$$\begin{aligned} & S=\sum\limits_{i=2000}^{2020}\sum_{j=i+1}^{2021}\text{sgn}\left(\text{GPR}{\text{I}}_{\text{j}}-\text{GPR}{\text{I}}_{\text{i}}\right),\\ & Z=\frac{S}{\sqrt{\text{Var}\left(S\right)}}, \end{aligned}$$

$$\text{Var}\left(S\right)=\frac{n\left(n-1\right)\left(2n+5\right)-{\sum}_{t=1}^{m}{t}_{i}\left({t}_{i}-1\right)\left(2{t}_{i}+5\right)}{18}$$

where:

$$\text{GPR}{\text{I}}_{\text{i}}\text{ is the GPRI value in year}i,$$

$$n:\text{number of years,}$$

$$m:\text{number of tied groups,}$$

$$\text{}{t}_{i}\text{:size of the }i\text{-th tied group,}$$

$$\text{sgn}\left(x\right)=1\text{\:if\:}x>\text{0,0}\text{\:if\:}x=0,-1\text{\:if\:}x<0.\:$$ [59, 60].

### Appendix III – Theil-Sen estimator

$$\begin{aligned} & \:\text{Theil-Sen slope}\; \widehat{\upbeta}=\text{median}\left(\frac{\text{GPR}{\text{I}}_{\text{j}}-\text{GPR}{\text{I}}_{\text{i}}}{{t}_{j}-{t}_{i}}\right) \\ & \quad \text{for all}1 \le i < j \le n, \end{aligned}$$

where:

$$\text{GPR}{\text{I}}_{\text{i}}\;\text{and GPR}{\text{I}}_{\text{j}}\text{:GPRI values at years }{t}_{i}\text{ and }{t}_{j},$$

$$n:\text{\:number\:of\:years}.$$ [65, 66].

### Appendix IV – Kendall’s Tau

$$\begin{aligned}{\uptau\:}=\frac{2}{n\left(n-1\right)}\sum_{i < j}\text{sign} \left(\text{GPR}{\text{I}}_{\text{j}}-\text{GPR}{\text{I}}_{\text{i}}\right) \end{aligned}$$

where:

$$\text{GPR}{\text{I}}_{\text{i}}\text{ and GPR}{\text{I}}_{\text{j}}\text{: GPRI values at years }i\text{ and }j,$$ $$n\text{:number of years,}$$

$$\text{sign\:returns\:}+\text{1,0},-1$$ [69]. 

### Appendix V – Moran’s I.

*Global Moran’s I is defined as* [73, 74] :

$$\:I=\frac{n}{W}\frac{{\sum\:}_{i}{\sum\:}_{j}{w}_{ij}\left({x}_{i}-\stackrel{-}{x}\right)\left({x}_{j}-\stackrel{-}{x}\right)}{{\sum}_{i}{\left({x}_{i}-\stackrel{-}{x}\right)}^{2}}$$

*Local Moran’s I is calculated for each unit i as* [73, 75]:

$$\begin{aligned} & {I}_{i}=\frac{\left({x}_{i}-\stackrel{-}{x}\right)}{{m}_{2}}\sum_{j}{w}_{ij}\left({x}_{j}-\stackrel{-}{x}\right),\\ &{m}_{2}=\frac{{\sum}_{i}{\left({x}_{i}-\stackrel{-}{x}\right)}^{2}}{n} \end{aligned}$$

where

$N\;$*number of spatial units (municipalities)*,

$W\:=\:{\sum\:}_{i}\:\sum\:_{j}{w}_{ij}:\:$*sum of all spatial weights*,

${w}_{ij}:\:$spatial weight between units i and j

${x}_{i}:\;$observed value (e.g., the GPRI trend statistic such as Theil–Sen slope),

$\overline{x}:$ the mean across all units.

## Data Availability

The datasets generated and/or analysed during the current study are not publicly available because they contain sensitive information subject to data protection regulations. Specifically, (i) the dataset on office-based physicians in Lower Saxony for the period 2000–2021 is protected under data protection law, and (ii) ATKIS data are likewise subject to data protection restrictions. However, aggregated and anonymized versions of the data may be made available from the corresponding author upon reasonable request and with sufficient justification.

## Data availability

The datasets generated and/or analysed during the current study are not publicly available because they contain sensitive information subject to data protection regulations. Specifically, (i) the dataset on office-based physicians in Lower Saxony for the period 2000–2021 is protected under data protection law, and (ii) ATKIS data are likewise subject to data protection restrictions. However, aggregated and anonymized versions of the data may be made available from the corresponding author upon reasonable request and with sufficient justification.
